# Mass Spectrometry Imaging for Spatial Chemical Profiling of Vegetative Parts of Plants

**DOI:** 10.3390/plants11091234

**Published:** 2022-05-02

**Authors:** Akhila Ajith, Phillip J. Milnes, Giles N. Johnson, Nicholas P. Lockyer

**Affiliations:** 1 Department of Chemistry, Photon Science Institute, University of Manchester, Manchester M13 9PL, UK; akhila.ajith@postgrad.manchester.ac.uk; 2 Syngenta, Jeolott’s Hill International Research Centre, Bracknell RG42 6EY, UK; phillip.milnes@syngenta.com; 3 Department of Earth and Environmental Sciences, University of Manchester, Manchester M13 9PY, UK; giles.johnson@manchester.ac.uk

**Keywords:** mass spectrometry, plant, chemical imaging

## Abstract

The detection of chemical species and understanding their respective localisations in tissues have important implications in plant science. The conventional methods for imaging spatial localisation of chemical species are often restricted by the number of species that can be identified and is mostly done in a targeted manner. Mass spectrometry imaging combines the ability of traditional mass spectrometry to detect numerous chemical species in a sample with their spatial localisation information by analysing the specimen in a 2D manner. This article details the popular mass spectrometry imaging methodologies which are widely pursued along with their respective sample preparation and the data analysis methods that are commonly used. We also review the advancements through the years in the usage of the technique for the spatial profiling of endogenous metabolites, detection of xenobiotic agrochemicals and disease detection in plants. As an actively pursued area of research, we also address the hurdles in the analysis of plant tissues, the future scopes and an integrated approach to analyse samples combining different mass spectrometry imaging methods to obtain the most information from a sample of interest.

## 1. Introduction

Understanding the physiological processes and associated molecular changes in plant systems is of paramount importance in numerous fields of studies associated with plants. Deciphering the molecular compositions of plants in a reliable, reproducible and precise manner can help uncover metabolic changes associated with growth, structure, stress, whole-plant resource allocation, plant–environment interactions, xenobiotic metabolism, etc. [1,2]. Metabolism in plants usually refers to endogenous metabolites involved in inherent physiological processes in plants. However, it is not limited to these, as xenobiotic compounds absorbed into the plant often undergo molecular degradation and modification in the plant systems. Plants produce an enormous wealth of primary and secondary metabolites as a result of the biochemical processes occurring within their systems and several methodologies are available to understand and analyse these. The metabolic composition of a system can be investigated in two ways, either in an untargeted fashion or in a targeted manner where the identity of the target molecule is known. Hence, the method adopted for chemical analysis may vary with the needs of the experiment. 

Generally, for any kind of metabolic investigations, the most sought-after methods include the different kinds of mass spectrometric approaches such as gas chromatography-mass spectrometry (GC-MS), liquid chromatography-mass spectrometry (LC-MS) and capillary electrophoresis-mass spectrometry (CE-MS) alongside nuclear magnetic resonance (NMR) spectroscopy [3]. Although these methods are well-established and can shed light on the molecular compositions and aberrations in a system of interest, the spatial information associated with localisation and movement of the biochemicals is unavailable as most of these techniques use homogenised samples. An insight into the spatial localisations of different chemicals in the plant system can be instrumental in understanding the movement and localisation of plant biochemicals and xenobiotics as well as their responses to different stress and metabolic pathways [4]. Fuelled by such motives, there has been a growing interest in the spatial profiling of metabolites over the past years in plant biology and this has been mostly achieved by immunohistochemistry, fluorescence microscopy or in situ hybridisation [5]. However, the experiments conducted using such techniques are limited to the visualisation of very few specific chemicals. In addition to the above-mentioned techniques, autoradiography is prominently used in agrochemical research to understand the uptake and translocation of xenobiotics in plant systems. Even though this technique is sensitive and produces quantifiable results [6] with reported spatial resolutions as low as 100 µm [7], there are several limitations including the time to synthesise radiolabelled compounds, along with the cost and safety concerns. In addition to these constraints, autoradiography does not discriminate between the chemicals of interest and their metabolites in the plant systems, as images are generated concerning all molecules, showing the radiolabelling and they do not detect those lacking the radioisotope. 

Mass spectrometry imaging (MSI) is gaining popularity as a mainstream method for spatial metabolic profiling as it addresses the limitations of these commonly used techniques to a large extent. Technical and methodological advances in the capabilities of MSI techniques in the past two decades make it possible to detect a wide array of biochemicals from small molecules to large proteins with high spatial resolution. In many cases, minimal sample preparation conserves the spatial information and increases the throughput of the experiment. The most popular MSI techniques currently in use are matrix-assisted laser desorption ionisation MSI (MALDI MSI), desorption electrospray ionisation MSI (DESI MSI), secondary ion mass spectrometry imaging (SIMS imaging) and laser ablation electrospray ionisation MSI (LAESI MSI). The reliability of MSI data in chemical analysis is evident from its application in studies associated with disease diagnosis [8,9,10], forensics [11,12], food analysis [12,13,14,15], analysis of environmental pollutants [16,17] and plant metabolite analysis. According to the needs of the experiment, the ideal MSI techniques can be chosen considering the spatial resolution and range of detection of analyte concentration and targeted mass-to-charge (*m*/*z*) values [18,19]. This review aims to explore the recent advances in the application of different mass spectrometry imaging modalities MALDI, DESI, SIMS and LAESI for imaging the natural plant metabolites, agrochemicals and disease detection in plant vegetative systems.

## 2. Mass Spectrometry Imaging

**Figure 1:**
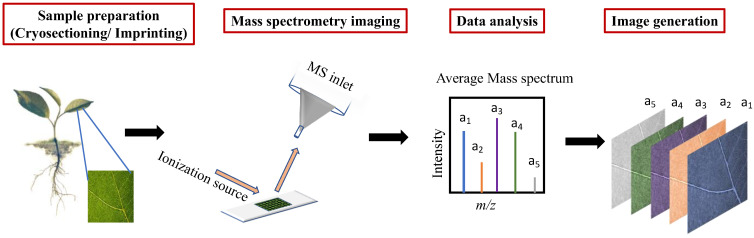
General workflow in an MSI experiment of plant tissues. A suitable sample (for example, a leaf section) from a plant is collected and prepared. With the aid of MS instruments such as MALDI, DESI, SIMS and LAESI with varying ionisation sources, the surface can be mapped for the constituent chemicals. The mass spectra obtained from each point in the sample along with the positional information can be used to create several ion images showing localisation for numerous chemicals of interest from a single scan.

In a typical MSI experiment, the workflow is as shown in Figure 1. The sample/plant species for an experiment is taken, e.g., leaf, root or stem section. If the experiment involves the application of any external chemicals (e.g., agrochemicals), specific methodologies are devised for this purpose. Samples are then collected and prepared as detailed in Section 2.2. The prepared samples are then taken for mass spectrometry imaging studies wherein a mass spectrum is collected from each point on the region of interest in the sample, such that every pixel in the image corresponds to one mass spectrum. The large collection of spectra obtained, along with their spatial information can be used to understand the localisation and distribution of various biochemicals present in the samples. Often advanced data analysis routines are applied to help interpretation (see Section 2.3).

### 2.1. Ionisation Sources for MSI

The choice of an MSI technique for a scientific investigation of any sample determines several factors, of which the most prominent ones are the spatial resolution, range of molecules detectable and the type of sample preparation required. Even though there are several MSI methodologies available, in consideration of the above factors, the most common MSI technologies in use are MALDI, DESI, SIMS and LAESI, each with its specific sample preparation, range of detection and resolution (Table 1). 

Based on the number of research papers, MALDI is the most popular technique for MSI [20]. As the name suggests, MALDI involves a matrix-coated surface that is subjected to laser pulses for ionisation. Matrix species and analyte molecules are co-desorbed and ionised by a mechanism which is still the subject of active research, generally involving proton/energy transfer between co-desorbed species resulting in ionised analyte molecules. MALDI is a soft ionisation technique as it imparts little residual energy to the target analyte hence resulting in a low rate of fragmentation of molecules. Although mostly performed under high vacuum, low vacuum and atmospheric, (AP-)MALDI sources are also available, including a high-performance atmospheric-pressure scanning MALDI microprobe ion source to obtain high-resolution ion images with the ability to image metabolites in the spatial resolution of a few microns [21]. More typically, the resolution of reported MALDI images is in the range 5–50 µm [22,23]. Atmospheric pressure MALDI widens the range of analytes and matrices to include volatile compounds, although often at the cost of ion transmission. Whereas UV lasers are most commonly used for MALDI, coupled to aromatic chromophores in matrix molecules, IR lasers can be applied to hydrated samples using the native water content as a matrix for laser absorption. However, IR lasers are more difficult to focus on small spot sizes and are less commonly applied in MSI studies. The wide detection capabilities of the MALDI technique is impressive, with the ability to detect small metabolites to large proteins [24,25]. 

SIMS was the first MSI methodology to be developed and uses focused ion beams to sputter surface chemicals under high vacuum conditions [26]. In biomolecular SIMS studies, the primary ion beam is typically composed of atomic or molecular clusters such as C_60_^+^, (H_2_O)^+^_n_ or (Ar_1000_)^+^ [26]. These polyatomic primary ions can produce ‘secondary’ analyte ions in a soft ionisation mode with 1–10 μm spatial resolution. With molecular depth profiling capabilities, 3D imaging is possible with modern SIMS machines [27]. Atomic primary ion beams (Ga^+^, Cs^+^, etc.) provide up to 50 nm spatial resolution for atomic secondary ions and molecular fragments [28]. The capabilities of SIMS makes it possible to analyse subcellular metabolomics with high spatial resolution and sensitivity [29].

In contrast to MALDI and SIMS, DESI is an ambient ionisation technique whereby the samples can be analysed under atmospheric pressure conditions with little or no sample preparation. Analysis with DESI is performed with an impinging spray of charged solvent microdroplets which wets the surface and desorbs later upon the arrival of subsequent droplets, forming secondary droplets, carrying with it the surface chemicals towards the MS inlet. The ambient ionisation, minimal sample preparation and the nearly undamaged surface of samples after imaging make DESI MSI ideal for in situ analysis of biological samples and there have been several studies that have explored this aspect of DESI [30,31]. The spatial resolution for a DESI MSI experiment generally ranges from 150 to 200 μm but with the relatively new nanoDESI developments, a spatial resolution of around 10 μm has been reported [32]. 

LAESI is yet another ambient ionisation method in which a mid-IR laser ablates the surface and produces particles which are then ionised on interaction with charged solvent droplets. The IR-laser applied is resonant with the absorption of liquid water (wavelength 2.94 μm) so is well-suited to hydrated samples such as plant vegetative parts. Similar to DESI, the ambient nature of imaging and the minimal sample preparation makes it a preferred MSI method for surface analysis in near-physiological conditions. The typical spatial resolution for LAESI is around 200 μm but a resolution of 40 μm has been recently observed by Taylor et al. with a ‘LAESI microscope’ to image single cells [33]. An interesting feature of LAESI is its ability to perform 3D imaging in ambient conditions [34], which is not possible with MALDI, SIMS or DESI.

Although the scope of this review is limited to the most widely-used MSI techniques for plant imaging, i.e., MALDI, DESI, SIMS and LAESI, several other MS methodologies have also been applied, including laser ablation inductively coupled plasma mass spectrometry (LA-ICP-MS) [35], colloidal graphite assisted laser desorption ionisation mass spectrometry (GALDI) [36] and liquid extraction surface analysis (LESA) [37], etc.

### 2.2. Sample Preparation

Sample preparation involves steps from procurement of the sample until it is ready to be analysed by analytical techniques such as mass spectrometry imaging. Sample preparation of plant tissues for MSI has been reviewed by Dong et al. [38]. The main plant parts of interest in this review are the leaf, stem and roots, and due to the differences in the surface characteristics, each of these has slightly different preparation strategies for MSI. Although, the general principles of MSI analysis remain similar for other plant parts such as fruits [39], seeds [40], flowers [41], etc. If xenobiotic metabolites are of interest, specific strategies need to be adopted to apply the xenobiotic chemicals before the preparation of plant parts for imaging.

When targeting endogenous plant metabolites, the sample preparation mainly involves storage and preparation of surfaces for imaging with cryo-sectioning and pre-treatments. Samples are stored at −80 °C before and after sectioning onto suitable substrate slides. Freezing quenches metabolic processes and storing at such temperatures for up to 1 year have not shown any degradation [42]. Delicate samples which might undergo degradation such as certain leaves can also be stored after imprinting them on Teflon (PTFE) or thin-layer chromatography (TLC) plates [43]. Samples are more easily sectioned by embedding in a suitable medium such as optimal cutting temperature (OCT) compound, carboxymethyl cellulose (CMC), gelatine, ice or their combinations. In a comparative study with leaf tissue, Li et al. observed that among the many commonly employed embedding media, gelatine gave the most favourable result with no delocalisation observed in specimens embedded in it [44]. Ice can also be used for embedding but the delocalisation of water-soluble chemicals can be an issue. OCT is not recommended for MSI methods due to the interference with analytes and ion suppression [45]. The preparation of hydrated tissue samples is often done by cryo-sectioning, wherein the samples are flash-frozen and sectioned at around −20 °C. For dry samples such as those obtained from certain plant stems, sectioning can also be done at room temperature conditions [46]. Sample sections are then mounted onto slides generally using conductive double-sided adhesive tapes or by thaw mounting. 

For stem and root tissues, the sectioning and fixation of tissues are quite straightforward as outlined above. However, for leaves, due to the presence of the cuticular waxes, the surface chemicals are often inaccessible by direct imaging with soft-ionisation MSI methods such as MALDI and DESI, and special sample preparation methods are needed to circumvent this issue. Removal of cuticular waxes can be done by treating with chloroform or chloroform/dichloromethane mixtures [47], by physical removal of the surface wax of the leaves by scrapping [48] or by stripping the epidermis [47]. However, when chemically treated, the surface delocalisation of chemicals can happen, causing signals to be unreliable. The physical removal of surface waxes may also affect the spatial resolution of the experiment [36]. An alternate option to these methods is imprinting, wherein the surface to be analysed is pressed against surfaces such as porous Teflon [49] or TLC plates [50] with a vice to extract the chemicals in the tissue. This can be done with or without heating or solvent extraction [43]. However, imprinting is restricted to relatively ‘fleshy’ plant tissues and smearing can happen during the process, thus limiting the spatial resolution [51]. In a recent study, Wu et al. used a nanoparticle immersed paper for imprinting in an LDI MSI study [52]. The Au nanoparticle on the surface acted both as a matrix and an imprinting medium to analyse xenobiotics and as a result, gave better desorption efficiency for the analytes. In addition to the above-mentioned sample preparation methodologies, MALDI involves an additional step for the application of matrix when compared to the other MSI techniques. The choice of the matrix for an experiment largely depends on the chemical properties of the analytes of interest. The most commonly used matrices in MALDI imaging of plant tissues are 2,5-dihydroxybenzoic acid (DHB), alpha-Cyano-4-hydroxycinnamic acid (CHCA), 1,8–bis (dimethyl-amino) naphthalene (DMAN), 9-aminoacridine (9-AA), sinapinic acid (SA), etc. [53,54]. Alternatively, nanoparticles can be used as a matrix as shown by Shiono et al. in an interesting study where Fe nanoparticles were used as a matrix to image plant hormones in rice roots [55].

For MSI experiments analysing xenobiotics, mainly agrochemicals, in addition to the general factors affecting the resolution and sensitivity of the experiment, the method of application of them onto the plants also plays a major role in the outcome of the imaging experiment. Generally, the agrochemicals are applied either by spraying onto the leaves to understand uptake through surfaces, [56,57] or through hydroponic systems to understand the shoot uptake [58,59]. A field application study can also be performed to understand the uptake and distribution of agrochemicals in a practical setting. For understanding leaf uptake in a practical setting, a track sprayer system can be used by calibrating the pressure, volume sprayed and speed of movement as demonstrated by Annangudi et al. [56].

### 2.3. Data Processing

After the imaging experiments are performed, large data sets containing information about the spatial localisation of various chemical compounds are obtained as hundreds or thousands of mass spectra. The large amount of data obtained is often integrated with the help of modern software such as SCiLS labs (https://scils.de/), BioMap (http://ms-imaging.org/biomap/) and MSireader (https://msireader.wordpress.ncsu.edu/) to name a few, and/or with MATLAB or Python routines. In a targeted experiment, the analyte is known and with the aid of tandem mass spectrometry experiments, confident molecular assignment of the ion signal can be ascertained with the aid of authentic reference materials or chemical standards. However, things are much more complex when looking at the data of an untargeted MSI experiment, which usually involves the comparison of a test and a control sample set. In such a case, the manual comparison of the data can be done, and this approach has been in practice for a long time, albeit tedious and subject to human error. Modern machine learning approaches [60,61] and deep learning [62,63] are attracting more attention in fields such as MSI. Multivariate data analysis tools including principal component analysis (PCA) [64,65], linear discriminant analysis (LDA) [66] and least absolute shrinkage selection operator (LASSO) [67] can reveal the ‘hidden’ mass spectral features that differentiate samples or regions of interest. Once the significant ion signals are identified, the next step is to determine their molecular identity. High mass resolution or tandem mass spectrometry data can be interpreted with the use of databases such as MassBank [68] or lipidMaps [69], etc. 

As with other MS methods, MS imaging can also be used (with care) for the quantification of constituent analytes in a sample. Unlike in more conventional GC-MS or LC-MS methodologies, MSI involves the analysis of surface compounds in a sample without any chromatographic separation and often without any sample clean-up. Hence the same strategies used in XC-MS involving bulk samples cannot be directly applied to MSI. Accurate quantification of the constituents in a sample requires the careful consideration of extraction efficiency and mass-dependent ion loss during analysis along with the matrix-dependent ionisation efficiency of desorbed surface species [70]. Although relative quantification is the most commonly used method, absolute quantification can also be performed with MSI but is often more tedious due to the analyte being present in a complex mixture on the surface of the sample. Normalisation to the total ion current or a standard [71] or the use of a tissue extinction coefficient [72] over a region of interest for normalisation of endogenous compounds can be used to compensate for the matrix effects in relative quantification. The commonly used experimental quantification strategies are on-tissue spotting of standards or the use of mimetic models [73]. However, when performing experiments for absolute quantification, an extra step of determination of the extraction efficiency from different tissues need to be calculated. Absolute quantification is straightforward if the extraction efficiency is known [74], although, it depends on several factors including the thickness and type of tissue section, chemical properties of the solvent used for extraction and the analyte, the scan rate and the spatial resolution used for the experiment. Advanced data processing has also been proposed to mitigate the main issue of signal variability caused by matrix effects and extraction efficiency in quantification studies [75].

## 3. Mass Spectrometry Imaging of Plant Vegetative Parts

### 3.1. Endogenous Compounds

The ability of MSI technologies to image a multitude of chemical species at the same time makes it a valuable approach to understanding the chemical localisation in plant systems. The availability of high spatial resolution information along with the quantitative and qualitative data for a broad range of chemical species is unparalleled for MSI in comparison to the other contemporary technologies. The following sections focus on the applications of common MSI techniques to image endogenous metabolites in plant leaves, roots and stems.

#### 3.1.1. MALDI Imaging Studies

The first application of MSI to analyse vegetative plant parts was to image agrochemicals in soybean leaf [59] at the start of the 21st century. Since then, the number of studies utilising MSI in plant science has been steadily increasing year after year [4]. In one of the initial studies, MALDI was used to image the distribution of water-soluble oligosaccharides in the stems of *Triticum aestivum* (wheat). Water-soluble oligosaccharides were being investigated as a potential indicator for grain yield [76] and the positive ionisation mode MALDI detected the potassium adducts of a range of oligosaccharides up to Hex_11_ with α- CHCA matrix. 

**Figure 2:**
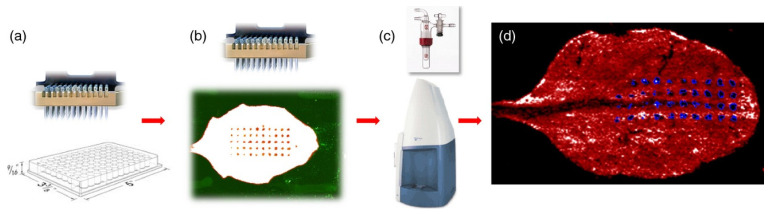
Quantification of glucosinolates on the surface of *A. thaliana* leaves. For the quantification study with MALDI-MSI: (**a**) A solution of internal standard (2-propenylglucosinolate) was mixed with a fluorescent dye and transferred with a pin array spotter to the leaf surface; (**b**) The quality of spotting was checked by a fluorescence scan; (**c**) The spotted leaves were covered with 9-aminoacridine matrix by sublimation and measured by MALDI-TOF mass spectrometry in the negative mode; (**d**) Collected data were analysed with Biomap software. Figure reproduced with permission from Shroff et al. [79].

Being a very important model species to understand plant physiology [77], there have been numerous studies with MSI to understand the chemical localisation of compounds in *Arabidopsis thaliana*. An early MALDI MSI study was performed to assess the feeding patterns of lepidopteran larvae in *A. thaliana* leaves [78]. The spatial distribution of glycosinolate, a compound involved in the plant defence [77], was assessed in negative ion mode MALDI with a 9-AA matrix. The observation of an abundance in glycosinolates in the midvein and periphery of leaves compared to other parts of the leaf, validated with HPLC analysis, led the authors to conclude that the preferential localisation of these compounds has profound roles in plant defence mechanisms. This study demonstrated the usefulness of MSI in understanding the chemical distributions of compounds to access different situations and phenomena associated with the plant system. Following this study, the authors did another investigation on the same plant species to develop a new MALDI imaging protocol in negative ion mode to image only the surface glycosinolates in the leaves [79]; the major difference in the protocol being that the 9-AA matrix was deposited now by sublimation rather than with an airbrush. In addition to imaging, surface quantification was performed with the aid of standards and it was found that the abaxial surface of the leaves contained about 50 pmol/mm^2^ surface area of the leaf with the adaxial surface having about 15–30% less (Figure 2). In contrast to the earlier results mentioned before, here, the surface distribution was relatively uniform with more abundance in the midrib and periphery of the leaf. LAESI and liquid extraction surface analysis (LESA) helped the authors to validate the distribution patterns observed. Another important class of molecules involved in plant defence mechanisms are the cyclic peptides, cyclotides which have insecticidal properties [80]. In an attempt to study novel cyclotide precursors in hybrid petunia leaves, MALDI was used in conjugation with genetic studies to identify and understand the localisations of potential cyclotide peptides [81]. MALDI imaging in positive ion mode with CHCA matrix identified 4 potential cyclotides with one being identified as Phyb A and the rest unidentified. The suspected species localised mostly in the vasculature of the leaf much akin to the potential roles in plant defence.

In another example with *A. thaliana*, the surface waxes on leaves were imaged with MALDI in both positive and negative ion mode with the lithium adduct of DHB as matrix [82]. Even though the laser irradiation time was longer than it would have been for polar target molecules, this study proved the ability of MALDI MSI to image neutral molecules such as wax esters and hydrocarbons in biological samples. The power of MSI in plant physiological experiments was also demonstrated in a study by Griffiths et al., using MALDI with CHCA matrix to visualise the effect of chemical intervention in the trehalose-6-phospate pathway in *A. thaliana* leaves [83]. With positive and negative ion mode MALDI studies, along with SIMS and plant physiological studies, artificial precursors to sugar were seen to increase grain size and total sugar production of the plant species. These results provide an alternate path to crop yield improvement through ‘biosynthetic amplification’ compared to the genomic methods.

Saccharides and surface waxes are essential to the survival of the plant system and are abundant in the plant biome, but the most abundant biopolymer present in the biological systems and in the world is cellulose [84]. The wide applicability of cellulose in paper manufacturing, food industry and as an alternative biofuel makes its spatial characterisation an important analytical problem [85]. For MALDI imaging of cellulose, DHB was found to be an ideal matrix by Jung et al., with microcrystalline cellulose standards compared to other popular matrices such as CHCA and sinapinic acid in positive ion mode [86]. The choice of matrix was also justified when the stem sections of *Populus deltoides* were imaged for cellulose. In another independent study with *P. deltoides* [87], MALDI with a linear ion trap for tandem MS measurements was used to image cellulose and hemicellulose in the stem sections in a more specific way, eliminating isobaric ions. In this study, too, with the help of microcrystalline cellulose and hemicellulose standards, DHB matrix was found to be the best for positive ion imaging compared to other matrices considered. 

Lipids in plants have storage functions, roles in plant defence and are signalling molecules [88], making the chemical mapping of lipid species in plants with MSI an important question for lipidomic investigations. In an early study to image lipids with MALDI, the lipid content of barley seed and tobacco root (*Nicotiana tabacum*) was assessed with HCCA and DHB as matrices in positive ion mode [89]. Lysophosphatidylcholines were identified and imaged, correlating with the data from standards and tandem MS measurements. An interesting investigation that utilised MALDI to image lipids was to understand the distribution of triacylglycerol in a transgenic model of *N. tabacum* leaves [90]. MALDI with DHB as matrix along with other mass spectrometry techniques such as GC-MS and tandem mass spectrometry revealed that the transgenic species of *N. tabacum* has a 15% more content of triacyl glycerol compared to the wild type. When comparing with the large biomass oil crops, such genomic changes can drastically affect the total oil yield relating to crops.

Root nodules in plants form as a result of a symbiotic relationship between the rhizobium bacteria and the plant to convert atmospheric nitrogen to ammonia for the plant [91]. An example of such a relationship exists between the plant *Medicago truncatula* and the rhizohium bacteria, *Sinorhizobium meliloti*. MALDI-MSI was used in an attempt to analyse the metabolic disparities in the root nodules formed by the symbiotic relationship [92]. With the complementary use of DHB and DMAN matrices in positive ionisation mode, the authors identified amino acids, organic acids, carbohydrates and flavonoids in the samples. Statistical data analysis performed with ClinProTools [93] showed that the root nodules contained higher glutamine and sucrose compared to the roots. In the 3D PCA plot, root and root nodule samples revealed very distinct distribution patterns implying distinct metabolic levels. In a subsequent study by the same group, the effect of salt stress on root nodules on the same species was studied [94]. In these experiments, in addition to MALDI wherein the system operated in a vacuum state, an atmospheric pressure MALDI (AP-MALDI) system was also used. The imaging experiments performed in positive ion mode were done with two matrices, DHB and CHCA, and were complemented by the tandem MS data of ion signals of interest. As a result of the Discriminative Analysis done with Receiver-Operating Characteristics (ROC), it was identified that control samples were enriched in asparagine, adenosine and nicotianamine, and the salt-treated samples in arginine and soyasaponin I. Even though AP-MALDI could achieve a higher resolution with smaller laser spot size, vacuum MALDI was able to detect significantly more *m*/*z* values.

Being sessile organisms, plants have developed several chemical mechanisms to cope with stress or situations of unfavourable growth conditions [95]. One such chemicals released in plants in response to biotic and abiotic stresses are the phytoalexins which are also known to induce antifungal activity [96]. When grapevine leaves were subjected to stress by exposure to UV-C radiation, MALDI in negative ion mode was able to find the peculiar localisations of phytoalexin compounds Resveratrol, Pterostilbene and Viniferins [97]. Although many matrices were tested, DHB gave the best results for imaging these phytoalexins. All three compounds of interest showed heterogenous distribution with co-localisations in the veins. In another study, Kaempferol-3-O-galactoside [trifolin + Na]+, a galactose-conjugated flavanol exhibiting antifungal and anti-cancer effects [97], was imaged in high abundance in the veins of the clover leaf with an atmospheric pressure MALDI MSI system [98]. With CHCA as matrix, the AP-MALDI could image these biological samples in atmospheric pressure conditions. 

**Figure 3:**
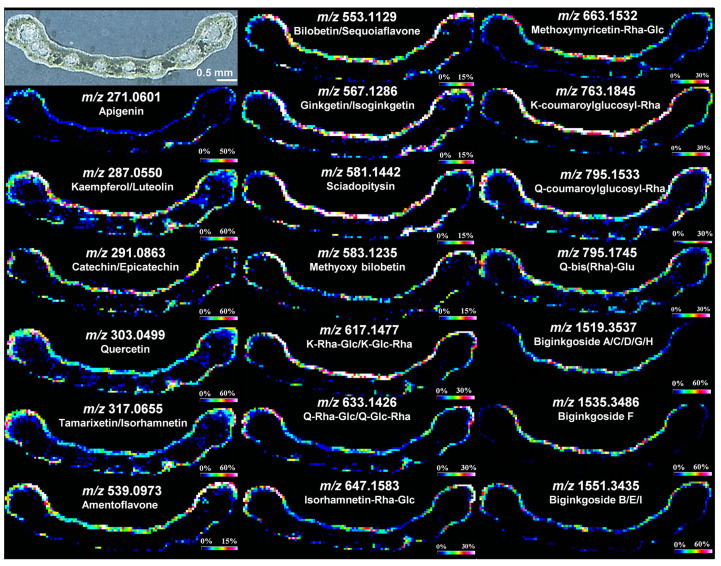
Cross-sections of ginkgo leaves imaged in positive mode MALDI showing images of selected flavonoid ions, including aglycones (*m*/*z* 271.0601–317.0655), biflavonoids (*m*/*z* 539.0973– 583.1235), glycosides (*m*/*z* 617.1477–795.1745) and biginkgosides (*m*/*z* 1519.3537–1551.3435) in ginkgo leaf. Ion images were recorded with a step size of 50 μm. The mass accuracy was less than 2 ppm and a bin width of *m*/*z* = ±5 ppm was used for image generation. Images represent the protonated, sodium and potassium adducts of metabolites. Glc: glucoside/glucosyl moiety; K: kaempferol; Rha: rhamnoside/rhamnosyl moiety; Q: quercetin. Figure reproduced with permission from Li et al. [101].

*Ginkgo biloba* is considered a ‘living fossil’ being the only extant member of the division of Ginkgophyta [99]. The roots, stem, leaves and seeds of *G. biloba* are a source of various bioactive metabolites, especially the leaves, which contains numerous secondary metabolites of pharmacological interest [100]. With MALDI and LDI MSI, Li et.al was able to image several metabolites on the surface of *G. biloba* leaves including rare flavonoid cyclodimers along with other substrates such as chlorophyll, phospholipids and saccharides [101]. The studies were utilised to obtain metabolic data in both positive and negative ion modes with CHCA and 9-AA as matrices, respectively. The relative quantification study done using selected flavonoids with LC-MS showed a slightly higher flavonoid content, in general, in the upper epidermis compared to the lower as seen in Figure 3.

Among the many factors affecting the spatial resolution of a MALDI MSI experiment, the spot size of the laser beam focus is very significant. In an attempt to achieve higher spatial resolution, Korte et al. adapted an instrument modification concept of Caprioli and co-workers [102,103], and modified the laser optics of a MALDI linear ion trap (LIT) Orbitrap mass spectrometer to image maize leaves in sub-cellular resolution with the use of 1,5-Diaminonaphthalene (DAN) matrix in negative ion mode [104]. The modified instrument allowed the detection and imaging of a variety of compounds including amino acids, ascorbic acid, phenolics, benzoxazinone derivatives, sugars and phosphate sugars, flavonoids and flavonoid glycosides and glycerolipids. Even though the achievable laser spot size was around 5 μm, for sufficient ion signals, 9 μm was considered a more practical laser spot size. In a subsequent attempt by the same group, a practical laser spot size of 5 μm was achieved by combining spatial filtering, beam expansion and reduction of the final focal length [105]. With maize root samples, the new MALDI system could image several molecules including phosphocholines and disaccharides in positive ion mode with DHB matrix. A feature of this system was that a user selectable laser spot size of ~4, ~7 and ~45 μm was subsequently achievable in about 5 min through an interchanging of the beam expander component. In another attempt to improve the resolution of MALDI MSI, Spengler and co-workers developed the atmospheric pressure scanning microprobe matrix-assisted laser desorption/ionisation mass spectrometer (AP-SMALDI) [21]. This novel system can image plant metabolites at cellular levels as demonstrated by Li et al. [106], where AP-SMALDI was used to analyse several plant secondary metabolites including gallotanins and monoterpene glucosides in *Paeonia lactiflora* roots. Another factor that affects the quality of images obtained with MALDI is the self-ionisation of matrix that interferes mostly with the ion signals in lower *m*/*z* values (~500 Da) [107]. For example, plant hormones are involved in signalling crosstalk and the basic physiological responses of cell [108], but being in the low mass range, many of them are not effectively detected with MALDI due to the chemical noise. With the introduction of Fe nanoparticles (Fe NPs) as the matrix, Shiono et al. [55] tried to circumvent this issue to image lower molecular mass plant hormones in positive ion mode in the leaves of the rice plant. When the results were compared with that of DHB matrix, nanoparticle assisted laser desorption ionisation (nano-PALDI) imaging was able to detect at least 4 more plant hormones and their precursors than MALDI.

#### 3.1.2. SIMS Imaging

The minimal sample preparation and the high spatial resolution are two of the factors for the increasing interest in SIMS for metabolic imaging, either used alone or in conjunction with other MS methods. Li et al. investigated the saccharides in switchgrass (*Miscanthus* *Χ* *giganteus*) roots with LDI MSI and SIMS imaging with gold primary ion source in positive and negative ion modes [109]. Several surface additives were used to check the signal improvements such as Au coating on samples and matrix enhancement with CHCA and DHB. The authors observed that in LDI MSI, a thin Au coating and DHB improved the signal intensity, and CHCA did not drastically affect the intensities. No one condition could image all the metabolites of interest and hence it required information from all the surface conditions for a complete overview. Unlike in LDI, the Au coating did not improve the signal intensities for SIMS and interestingly, it was seen that the non-coated section displayed higher quality images in negative than the positive ion mode.

In many trees, the central part of the wood is dark with a high density of organic solvent extractable compounds surrounded by lighter coloured sapwood. In Japanese cedar (*Cryptomeria japonica*), SIMS could identify exclusive ion signals of ferruginol, a diterpene phenol in high intensity in the heartwood tissue and not the sapwood tissue taken with a Ga^+^ ion beam in positive ion mode [110]. Although exclusive to heartwood, ferruginol was seen to uniformly distribute in the heartwood part of the wood tissue. In a following study by the same group, specific chemicals could be detected in the sapwood and heartwood-sapwood boundary in a 1500-year-old sample of Hinoki cypress (*Chamaecyparis obtusa*), helping to distinguish them in a visibly indistinguishable sample [111]. Using a Au^+^ primary beam in both secondary ion polarities, chemical substances hinokinin, hinokiresinol, hinokione and hinokiol were observed to be accumulated in the heartwood-sapwood boundary. Among these chemicals, only hinokinin was seen to accumulate in the ray parenchyma cells. This study is another example for the ability of SIMS in imaging even minute chemicals in biological samples such as wood tissue.

In another innovative study, Zhao et al. used a combination of Bi_3_^+^ SIMS with C_60_^+^ sputtering to image single-cell walls for the presence of syringyl (S) and guaiacyl (G) lignins in *Populus trichocarpa* wood tissue [46]. With positive ion mode imaging, the S-lignins were predominantly located in the fibre cell walls and the G-lignins in the vessel cell walls. The G/S-lignin ion intensity ratio in vessel cell walls was found to be double that in fibre cell walls, thus agreeing with the earlier study with UV microscopy which gave similar results [112]. In another study, G- and S-lignin levels were evaluated in the wood tissue of maple trees [113]. With Au^+^ ion beam in positive mode, the vessel walls were seen to be rich in G-lignin with varied S/G ratios through the growth ring in which the earlywood was seen to be rich in S-lignin and latewood had comparatively lesser. Both studies demonstrate the utility of SIMS in analysing the lignin composition in wood.

By providing the highest resolution among other mass spectrometry imaging techniques, SIMS is well-suited to analyse cellular localisation of metabolites and intermediates to understand the basic biochemistry. When SIMS was used to image γ-lactones and its intermediates in *Sextonia rubra* [114], some surprising observations were made contrary to the established metabolic pathways. SIMS in positive ion mode with Bi_3_^+^ ion beam for analysis and Ar_1000_^+^ for sputter could identify 5 γ-lactones including rubrynolide and rubrenolide along with the intermediates and their cellular localisation in the wood tissue samples. In light of the surprising new data obtained, the authors proposed a revised metabolic route for rubrynolide involving a reaction between 2-hydroxysuccinic acid and 3-oxotetradecanoic acid in place of an earlier biosynthesis with a single polyketide synthesis [115].

High resolution MSI provides huge data sets which, when the appropriate data analysis tools are used, can yield significant information. When SIMS was performed on the leaves of *P. trichocarpa* leaves, multivariate data analysis could detect chemical localisations and diverse patterns underlying the leaf surface [64]. The Bi_3_^+^ cluster ion beams in positive and negative ion modes detected several alcohols, hydrocarbons and wax esters in the epicuticular layer on the surface of the leaf similar to the MALDI data taken as reference, but with higher spatial resolution. For PCA, with 19 ions as variables, only a small number of principal components were sufficient to establish the maximum variability in the data. To complement the PCA data, when five-factors multivariate curve resolution (MCR) was done, distinct patterns of islets were apparent from the score plots. However, the authors recommend that the multivariate analysis, when done using cluster analysis, gives the best results as they showed more localisation and distinct chemical specificity.

#### 3.1.3. DESI Imaging

**Figure 4:**
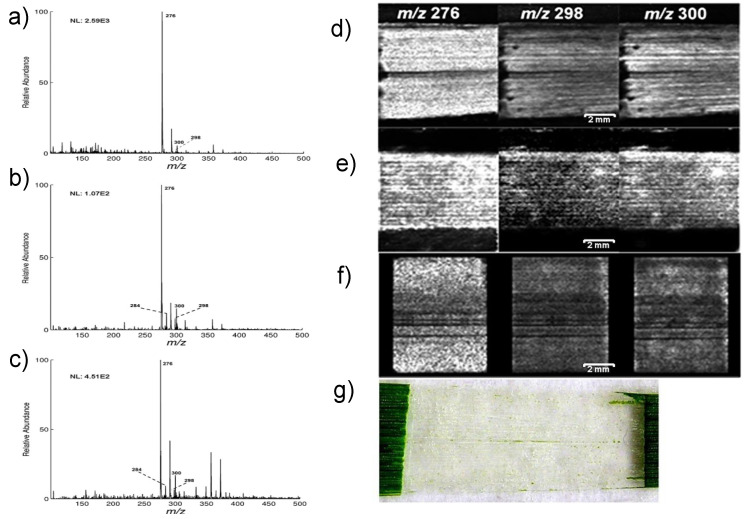
DESI MS spectrum and images of barley leaf under different sample preparation conditions: (**a**) DESI MS spectrum of a barley leaf (cv Mentor) treated with chloroform; (**b**) DESI MS spectrum of the Teflon imprint of the leaf epidermis; (**c**) DESI MS spectrum of the Teflon imprint of the intact leaf. DESI images are of the hydroxynitrile glucosides of *m*/*z* = 276, 298 and 300 from barley (cv Mentor); (**d**) Direct DESI images of the isolated epidermis; (**e**) Indirect DESI images of the isolated epidermis; (**f**) Indirect DESI images of the intact leaf; (**g**) Photo of the transparent leaf epidermis mounted on double-sided tape. All spectra were recorded in positive ion mode. The pixel size is 100 μm and the acquisition times were 120 min. Figure has been adapted with permission from Li et al. [47].

DESI being an ambient ionisation method, with minimal sample handling it offers the opportunity to image samples in near-physiological conditions in an efficient manner. As it is a soft ionisation method, many of the chemicals can be imaged as ions without much fragmentation. With no application of matrix, it also frees the investigation from matrix-related issues and concerns. One of the earliest studies to use DESI in plant sciences was by Thunig et al. wherein the leaves and petals of *Hypericum perforatum* were imaged in negative ion mode and the leaves of *Datura stramonium* in positive ion mode [49]. To overcome the hurdles posed by the waxy cuticular layer of the leaves, the imaging was done in an indirect manner with Teflon imprints of the leaf. Several secondary metabolites were imaged in the leaves and petals of *H. perforatum* including hyperforin and hypericin, and the leaves of *D. stramonium* revealed the presence of a few terpene alkaloids. Secondary metabolites are of great interest to many industries with plant-based raw materials. Such is the case with hydroxynitrile glucosides which are of interest due to their role in whiskey production [116] and its antifungal actions [117]. In a subsequent study by the same group, the hydroxynitrile glucosides in barley leaves were analysed with DESI MSI in positive ion mode [47]. Even though the hydroxynitrile glucosides are present on the epidermis of the leaf, direct imaging could not successfully image it and the effort to remove the cuticular waxes by chloroform treatment was not very efficient and reliable. However, the target signals were observed when analysis was performed in the peeled- off epidermis and in the Teflon imprints of the leaf, as seen in the mass spectra obtained shown in Figure 4a–c. Imaging of imprints proved to be an efficient way to analyse samples as seen by comparing Figure 4d–f. The ion signal abundances observed for different varieties of barley agreed with the corresponding LC-MS analysis. In the following study, along with visualisation, the enzymatic conversion of hydroxynitrile glucosides was also imaged in a Teflon imprint of *Lotus japonicus* leaf with indirect DESI MSI [118]. Hydroxynitrile glucosides are very sensitive phytochemicals that undergo degradation by specific β-glucosidases upon cell disruption [119]. Following damage to a restricted area in the plant, DESI was able to visualise the enzymatic degradation and localisation as well as the concomitant release of glucose. The effectiveness of DESI in both ionisation modes for metabolite visualisations was demonstrated with *L. japonicus* and was supported by an adjacent investigation in the cassava tubers in the same study. 

The de-greening or colour loss of leaves in autumn is a result of the seasonal degradation of chlorophyll in leaves [120]. The final product of this degradation process are the molecules polyfunctionalised nonfluorescent chlorophyll catabolites (NCCs) [121]. When the presence of the NCCs was investigated in the Katsura tree (*Cercidiphyllum japonicum*), American sweetgum (*Liquidambar styraciflua*) and hophornbeam (*Ostrya virginiana*) with DESI, the direct imaging did not produce sufficient signals in both ionisation modes [122]. However, when the leaf imprints were used, several NCCs showed higher signal intensities and could be identified and localised in a more efficient manner along with tandem MS and reactive DESI experiments using modified solvent sprays. The ability of the imprinting technique to image subtle features of leaves was demonstrated by Hemalatha et al. with *Catharanthus roseus* leaves and petals [50]. The secondary metabolites detected in this study in positive ion mode were able to image delicate features such as eye colour of petals, leaf vacuole, leaf margin and veins.

In efforts to improve the imaging capabilities of DESI MSI for plant parts, many studies have been conducted. For example, Li et al. tried a ternary solvent system of CHCl_3_–ACN–H_2_O (1:1:0.04) in place of the traditional binary solvent system such as CHCl_3_–ACN for direct DESI MSI [123]. This new solvent system, when tried on *Hypericum perforatum* leaves and petals in negative ion mode, DESI could image several very long-chain fatty acids (VLCFAs) and several other metabolites. When compared to the traditional binary solvent systems, numerous new metabolites could be visualised with this new system in the petals. The leaves also showed a better metabolic profile with the new solvent system than with the binary solvents. When treated with chloroform, even more metabolites could be analysed as the inner leaf layers were exposed. In indirect DESI MSI, among the many factors that control the quality of the image, blotting is a step of paramount importance. In an interesting study, to achieve improved transfer of metabolites to the surface of TLC plates, solvent extraction and heating was used independently and together for negative and positive ion mode DESI [43]. The novel blotting technique was tried on potato sprout (*Solanum tuberosum L.*), gingko leaves (*G. biloba* L.) and strawberries (*Fragaria x ananassa Duch.*). DESI MSI analysis could image glycoalkaloid toxins in potato sprout, ginkgolic acids and flavonoids in ginkgo leaves, and sugars and anthocyanidin in strawberries. The different samples taken in this study showed different optimal blotting conditions. Hence it was evident that the suitable blotting technique for an indirect imaging experiment depended on multiple factors such as the chemical property of the molecule of interest, chemical properties of the surface and that of the solvent when solvent was extracted. This technique of blotting with solvent and heating was also used in another study to image alkaloids in *Sassafras albidum* [124]. For improved separation and detection, the root, twig sections and leaves of *S.albidum* were analysed with high-performance thin-layer chromatography with multistage DESI MS along with DESI MSI. A total of 12 alkaloids were observed in the roots and twigs, of which 6 had not been detected before. Interestingly, all the alkaloids were localised around the root outer lines and no alkaloids were detected in the leaves.

#### 3.1.4. LAESI Imaging

For analysis, MALDI requires the application of a matrix to the sample surface and electrospray ionisation (ESI) requires samples to be in solution form, as imaging of biological samples in vivo in the native environment is not possible with these methods. LAESI, being an ambient technique with little or no sample preparation presents an alternative option along with DESI to image biological samples with sufficient water content [125]. 

**Figure 5:**
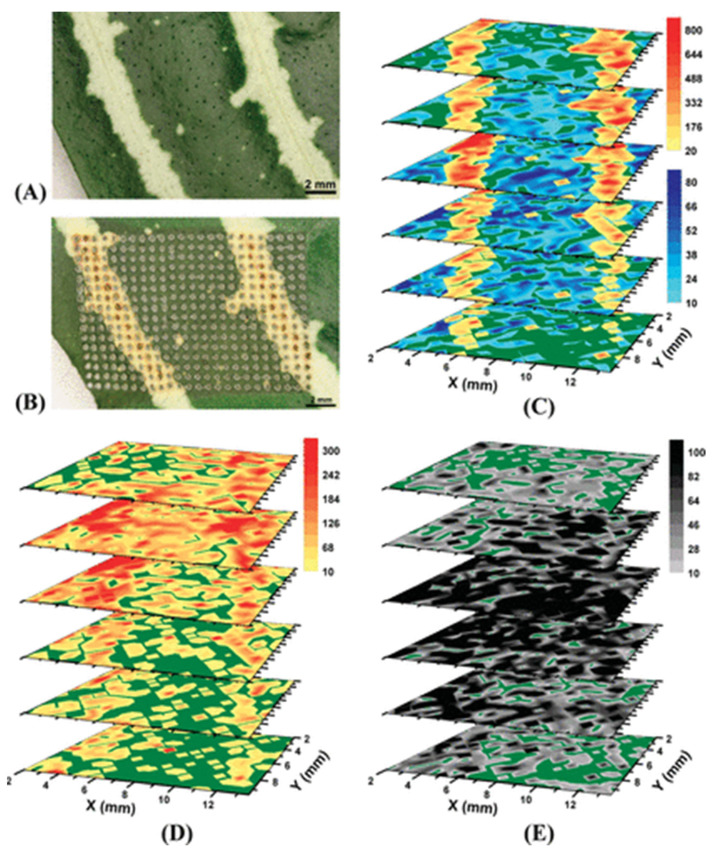
LAESI 3D imaging MS of *A.squarossa* leaf tissue where metabolites in relation to tissue architecture captured. Optical image of *A. squarrosa* leaves (**A**) before and (**B**) after analysis; (**C**) LAESI 3D imaging MS distribution of kaempferol/luteolin with *m*/*z* 287.0 (yellow/orange scale) followed the variegation pattern. Chlorophyll a with *m*/*z* 893.5 (blue scale) accumulated in the mesophyll layers; (**D**) Acacetin with *m*/*z* 285.0 showed higher abundance in the yellow sectors of the second and third layers with a homogeneous distribution in the others; (**E**) Kaempferol-(diacetyl coumarylrhamnoside) with *m*/*z* 663.2 accumulated in the mesophyll layers (third and fourth) with uniform lateral distributions. Figure reproduced with permission from Nemes et al. [34].

In one of the very first studies with LAESI, the variegation pattern of *A. squarrosa* leaf tissues was analysed to understand the metabolic differences in the green and yellow sectors of the leaf [126]. Several primary and secondary metabolites were visualised in positive ion mode including those involved in the biosynthesis of flavonoid kaempferol. A lateral resolution of ~350 μm and a depth resolution of ~50 μm was achieved in this study. However, an improved spatial resolution of ~300 μm and depth resolution of ~30–40 μm was achieved in a subsequent study by the same group in *A. squarrosa* and *Spathiphyllum lynise* leaf tissues [34]. In addition to the improved resolution, this study also reports the 3D imaging of leaf tissues with LAESI in positive ion mode. The accumulation of phytochemicals with respect to the variegation and their changing distribution with respect to the various cell layers of *A.squarossa* leaf is clearly visible in Figure 5A–E, adding to the results of the earlier study in 2D imaging [126]. Coupled with tandem mass spectrometry, the authors were able to image several secondary plant metabolites and found the tissue-specific distribution and accumulation patterns for them. The direct sampling and subsequent ionisation of surfaces by MSI techniques limits the differentiation of isobaric ions. Li et al. integrated ion mobility spectrometry to normal LAESI MSI so that molecules with the same *m*/*z* values could be separated and visualised [127]. To test this new system, frozen mouse brain tissue sections and leaf sections of *Pelargonium peltatum* were imaged in positive ion mode. The analysis of the abaxial surface of *P.peltatum* revealed the distributions of flavonoid glycoside ions.

### 3.2. Crop-Protection Products

Xenobiotics refer to any foreign chemical introduced to a biological system be it animal or plant [128]. The main xenobiotics of interest imaged in plants are agrochemicals or crop-protection products including insecticides, herbicides and pesticides. The field of crop protection product imaging with MSI is quite unexplored (Table 2) when compared to the wealth of studies done to image endogenous plant chemicals. However, the need to understand and image the distribution and localisation of various agrochemicals are becoming critical in the present times when a global food crisis is an imminent possibility [129]. 

Conventionally, autoradiography has been used to image the uptake and translocation of crop protection products in plant systems for agrochemical research [130]. In autoradiography, the radiolabelled compounds of interest are either applied in the foliage or mixed in the nutrient solution in hydroponic systems to study the foliar and shoot uptake, respectively [130,131,132]. Although widely used, the need for tedious synthesis of radiolabelled compounds and the ambiguity in distinguishing the principal compound and metabolites along with the safety concerns, necessitates the availability of an alternative approach to autoradiography. Mass spectrometry imaging provides such an option, being an untargeted screening method with a relatively easier and fast sample preparation methodology.

The earliest study with plants to image xenobiotics was done in soy plants to image the herbicide, Mesotrione, and the fungicide, Azoxystrobin with MALDI MSI [59]. For direct and indirect imaging (with blotting), the leaves were spotted with 1 μL droplets of Mesotrione (1.7 mg/mL) and Azoxystrobin (1.8 mg/mL) in 50:50 acetone/0.1% Tween20 and the samples were imaged at different time periods after application. Azoxystrobin was imaged in negative ion mode with SA matrix and Mesotrione in positive ion mode with CHCA matrix. Along with leaf surface detection for both the compounds, the authors also imaged the uptake of Azoxystrobin through the roots of the plants to the stem by spiking the growth medium until a concentration of 40 ppm was achieved with Azoxystrobin. Spiking individual plants growing in hydroponics with Azoxystrobin also enabled the shoot mobility analysis experiments which were performed 48 h after application. This showed that Azoxystrobin was indeed absorbed through the roots of the plant and was translocated through the stem. In a similar study Nicosulfron, a pyramidylsulfonyl urea herbicide, was imaged in sunflower plants with MALDI MSI in positive ion mode with CHCA matrix [57]. This study looked into both the foliar as well as the shoot uptake of the herbicide. For the shoot uptake experiments, the growth solution of the plant was spiked until a concentration of 40 ppm was reached, and for the foliar uptake, 1 μL aliquots of 1.25 mg/mL Nicosulfron in 50:50 acetonitrile/Tween was applied and sections varying in lengths from the plants were taken later for analysis. Interestingly, the major metabolites used for imaging in these experiments were formed by the breaking of the urea bonds in the substituted pyramidylsulfonyl urea herbicide. In a study by Annangudi et al. [56] on wheat leaf surfaces, MALDI MSI was used to detect 500 ng of commercial fungicides Epoxiconazole, Azoxystrobin and Pyraclostrobin in 1 µL drops on the leaf surfaces. The imaging studies done with DHB matrix in positive ion mode could even detect Pyraclostrobin in amounts as low as 60 ng in 1 µL droplets. The field application study performed also showed promising results as the fungicides could be visualised when applied at a field rate of 100 gai/ha in 200 L water using a track sprayer system. 

**Figure 6:**
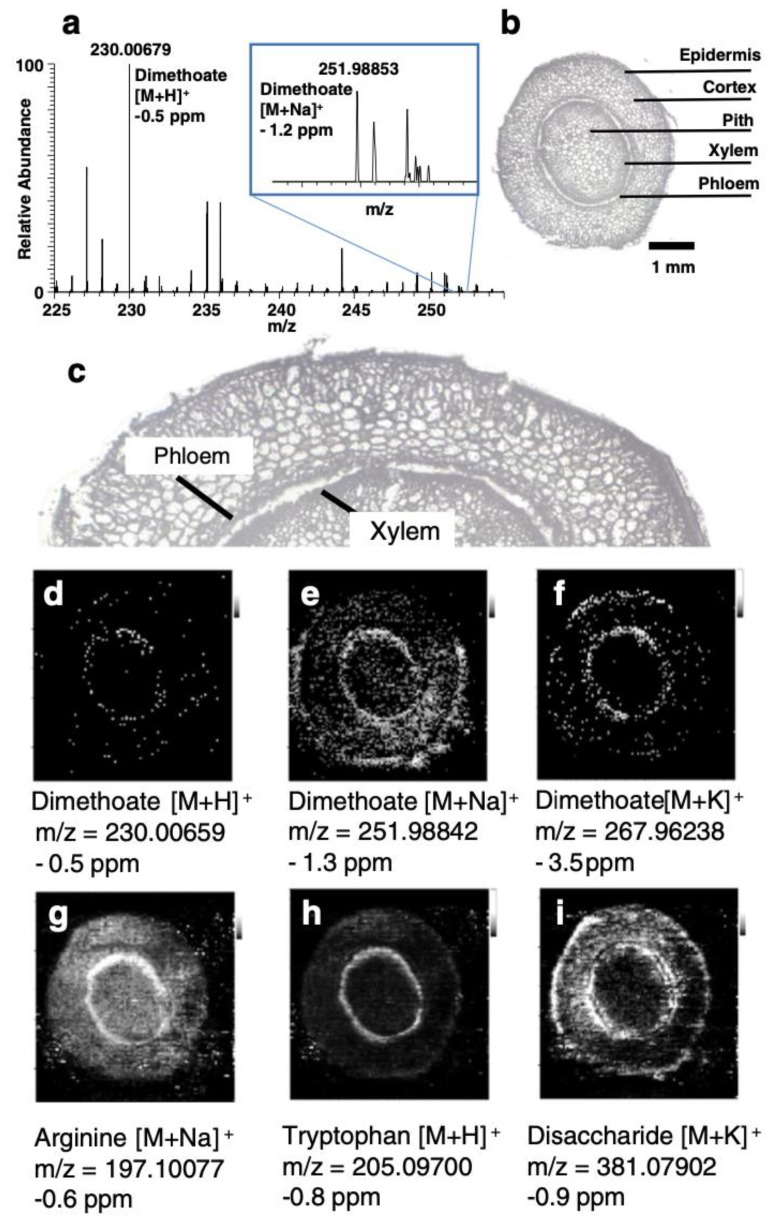
Images obtained from the dimethoate experiment on *Kalanhoe blossfeldiana* to see root uptake: (**a**) DESI mass spectrum of protonated dimethoate and sodium adduct detected in spiked soil; (**b**) Optical image of Kalanchoe stem cross-section; (**c**) Enlarged area of stem section to improve visualisation of xylem and phloem area; (**d**–**f**) Distribution of dimethoate in the plant stem, detected as protonated species, sodium and potassium adduct; (**g**–**i**) DESI-MS images of different naturally occurring substances. Pixel size of the DESI-MSI experiment was set to 60 μm. Figure reproduced with permission from Gerbig et al. [133].

Although most studies involving agrochemical compound imaging have been done with MALDI, researchers have also applied other MSI methodologies for imaging. An example is that of an investigation to analyse contact and systemic pesticides with DESI MSI in positive ion mode [133]. The authors considered two commercial contact insecticide sprays for the experiments on *Cotoneaster horizontalis*, one containing natural insecticides pyrethrin and rapeseed oil, and the second containing synthetic insecticides, Imidacloprid and Methiocarb. Even though both natural and synthetic contact pesticides were subjected to similar spraying methods and drying time of 30 min, the latter showed a more homogeneous distribution compared to the former. For the systemic insecticide experiment, dimethoate tablets were spiked into the soil to gain a concentration of 33 mg/kg of the soil *of Kalanchoe blossfeldiana* and were detected in the transport system of the plant in 25 days. The stem cross sections showing the distribution of associated ions can be seen in Figure 6d–f along with the associated MS spectra in Figure 6a. After 60 days of application of dimethoate, the presence of it was detected as a homogenous distribution in the leaves.

MSI can also be performed in conjunction with other analytical techniques to yield a more holistic result providing a broad wealth of information. The common methods usually used with MSI for multimodal imaging include vibrational spectroscopic methods including Fourier transform infrared (FTIR) and confocal Raman microscopy (CRM) [134], fluorescence microscopy [135], Liquid extraction surface analysis (LESA) [136], etc., to name a few. An example of this in the field of plant biology is a multimodal study to analyse the phenylamide fungicide Metalaxyl in tomato plants where LC-HRMS^n^ (liquid chromatography-high resolution accurate mass spectrometry), autoradiography and MALDI MSI with CHCA matrix in positive ion mode were used together to aid in identification and quantification [58]. In this study, with hydroponic systems containing 0.2 ppm Metalaxyl concentration, extensive metabolism of the parent compound was observed after 10 days in shoots and leaves but the parent compound was only detected in the roots. 

In another study, a novel probe design combining electrospray ionisation (ESI) and atmospheric pressure chemical ionisation (APCI) compatible with the LAESI instrument was introduced to study the translocation of fungicide Isotianil and its metabolite in tomato leaves [137]. Leaves were spotted with 10 µL of commercial fungicide formulation having a concentration of 250 ppm at the base of the leaf. The novel probe was seen to provide improved pixel to pixel repeatability for LA-APCI than the traditional LAESI in both ionisation modes. The applicability of this novel design was demonstrated by its ability to investigate the translocation of Isotianil and its metabolite, anthranilonitrile, showing the movement of anthranilonitrile from treated to untreated leaves. Innovation and novel strategies can also be adapted to improve the sample preparation methods. An example of a study, along these lines is by Wu et al. where a gold nanoparticle immersed paper was used for imprinting for laser desorption ionisation MSI (LDI MSI) to image a carrier-mediated form of the insecticide, chlorantraniliprole, in flowering cabbage leaves [52]. Generally, porous Teflon and TLC materials are used for imprinting [49,50], but low laser absorption and surface charge accumulation associated with these materials are often not ideal for laser-based MSI techniques. The flowering cabbage leaves were brushed with 10^−4^ mol/L of chlorantraniliprole and air-dried to prepare samples for imprinting. The nanoparticle imprinted paper seemed to show an improved ionisation efficiency while imaging the alanine ethyl ester-chlorantraniliprole conjugate (CAP-Ala), the carrier-mediated pesticide. With the aid of the nanoparticle imprinted paper, the authors observed that the carrier-mediated pesticide moved faster than the native pesticide towards the phloem and the primary vein. 

### 3.3. Disease and Pathogen Detection

**Figure 7:**
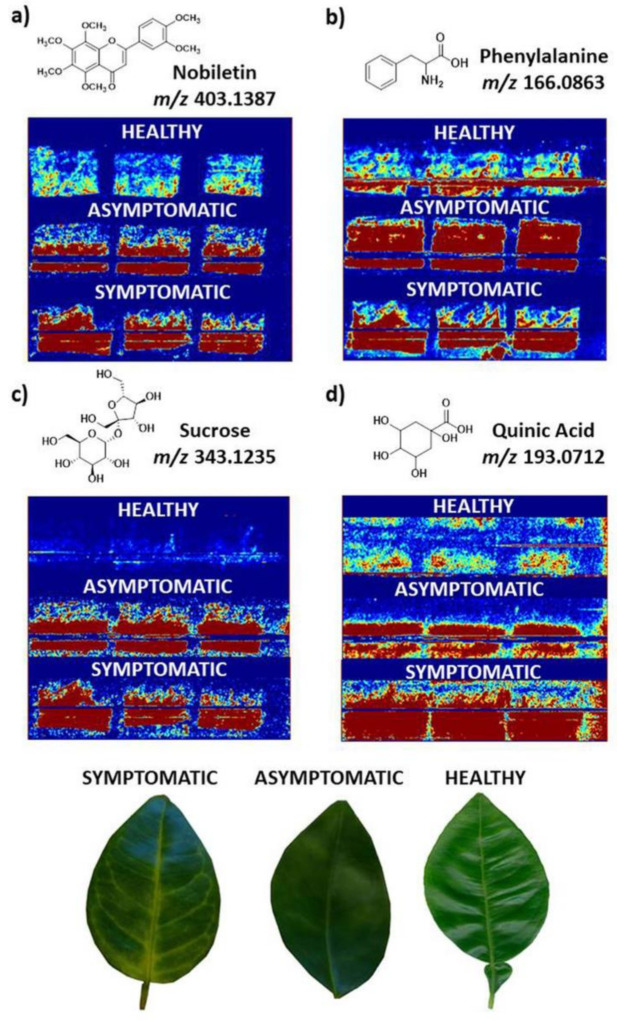
Images acquired for the various biomarkers identified for HLB disease with DESI MSI in positive ion mode. In the images acquired, the red colour indicates the ion distribution on the abaxial leaf surface in asymptomatic, symptomatic and healthy samples. The images acquired were at: (**a**) *m*/*z* 403; (**b**) *m*/*z* 166; (**c**) *m*/*z* 343 and (**d**) *m*/*z* 193, which were the exact masses found in Xcalibur software (*m*/*z* 403.1387, *m*/*z* 166.0863, *m*/*z* 343.1235, and *m*/*z* 193.0712) corresponding to nobiletin, phenylalanine, sucrose and quinic acid, respectively. Figure reproduced with permission from de Moraes Pontes et al. [48].

Similarly, as in humans and other organisms, the diseases affecting plants can also vary in the level of fatality. However, with plants being the primary source of food for the growing populations around the world, plant disease has great implications [138]. The cause of such diseases include fungi, bacteria, viruses, viroids, algae and many other pathogens [139]. Huanglongbing disease (HLB) is such an example of a plant disease affecting citrus species. Caused by the bacteria *Candidatus Liberibacter* spp through the vector *Diaphorina citri* or *Trioza erytreae* and by grafting [140,141], HLB disease can lead to the formation of defective, small lopsided fruits. Pontes et al. proposed MSI as an alternative diagnostic method for HLB disease with leaves in place of the generally used polymerase chain reaction [48]. Direct DESI MSI in positive and negative ion mode with tandem mass spectrometry could identify several metabolite classes and their variations characteristic of the disease. Among other metabolites which showed variations in the disease, the images showed an increased quinic acid, nobiletin, sucrose and phenylalanine in the diseased leaves when compared to healthy ones (Figure 7). The minimal sample preparation and fast and efficient imaging make DESI MSI a potential alternative to the traditional PCR analysis. 

Epiphytes are organisms that non-parasitically survive on the surface of the plants [142]. Even though non-parasitic, their presence can alter the metabolic composition on leaf surfaces. When analysed with a commercial ‘universal MALDI matrix’ in positive ion mode on *Arabidopsis thaliana* leaves, it was seen that after epiphytic colonisation, the surface sucrose, fructose and glucose levels were altered [143]. For the organoheterotroph, *Sphingomonas melonis* or the phytopathogen, *Pseudomonas syringae*, the levels of above-mentioned sugars were significantly altered, but for the methylotroph, *Methylobacterium extorquens*, changes were minor compared to the control. The alterations were also observed in the arginine metabolism and phytoalexin synthesis in the leaves hosting these micro-organisms. Interestingly, it was observed that the magnitude changes in the levels of characteristic metabolites were comparable to that usually seen in a mammalian host–microbe interaction [144].

Mycotoxins represent a class of fungal secondary metabolites with a pre-eminent role in plant infection [145]. MSI represents an ideal methodology to understand the complex biochemical responses of plants against such unfavoured xenobiotics [146,147]. Righetti et al. used high spatial resolution atmospheric pressure scanning microprobe matrix-assisted laser desorption/ionisation mass spectrometry imaging (AP-SMALDI MSI) to investigate the interplay between *Zea mays* (maize), the mycotoxin aflatoxin [148]. In response to the unfavourable xenobiotic presence in the system, the authors found that the anthocyanin and chlorophyll metabolism appears to be inhibited by the accumulation of aflatoxin in the maize roots. 

## 4. Concluding Remarks

Plant imaging with mass spectrometry is a rapidly developing field of wide significance. With the ability to image a multitude of chemicals in a sample with a single scan retaining the spatial information, MSI is an efficient and relatively fast alternative to the traditional metabolomic approaches. However, the issues related to sample preparation of leaves, quantification and the limit of detection are targets for current and further development in MSI as described in this article. This review focuses on the imaging of plant vegetative parts such as leaf, stem and roots. However, the mass spectrometry imaging in plants is not limited to these samples and the imaging of seeds [40,149], fruits [39,150] and flowers [41,50] are actively pursued along with that of the vegetative parts of the plant. MSI is also applied in environmental studies to understand various related mechanisms and processes such as the effect of pollutants and contaminants in ecosystems [17].

For quantification studies with MSI, matrix effects are one of the main challenges for determining the ionisation efficiency and limit of detection and need to be accounted for in accurate quantification. In MALDI MSI, matrix crystal size, laser focus restrictions, analyte delocalisation and degradation within matrix crystals and detector sensitivity are the major factors limiting the spatial resolution. Novel methodologies are being devised to improve MALDI imaging considering these issues [151]. In SIMS imaging, primary ion beams with higher energy, smaller spot size and softer ionisation capabilities to detect a wide range of analytes present the major areas of development [152]. Even though analysis can be conducted in atmospheric pressure conditions for ambient ionisation MSI techniques, the achievable spatial resolution, detection range of molecules, quantitative ability and sensitivity still are issues to be dealt with in techniques such as DESI and LAESI MSI [153]. One approach to enhance selectivity and efficiency in the ionisation step in electrospray-based methods is to include additives or reagents in the solvent spray, for example, in the methodologies termed ‘reactive DESI’ [154,155] and ‘reactive LAESI’ [156]. An alternative approach, for (MA)LDI and SIMS-based methods is to decouple the desorption and ionisation steps in order to provide routes to optimise each independently. Laser post-ionisation methods have been shown to offer considerable advantages in terms of analyte coverage and detection sensitivity [157,158,159,160]. In this approach, a laser beam is directed parallel to the sample surface to ionise the neutral fraction of the desorbed analyte plume, which typically dominates the desorbed ion fraction by several orders of magnitude. A promising approach for post-ionisation in atmospheric ion sources including AP-MALDI is the application of plasma sources [161].

With the capabilities of MSI imaging evolving in terms of resolution, sensitivity and depth profiling, the complexity of data is also increasing hence demanding robust, high throughput data analysis tools. Machine learning, deep learning and artificial intelligence present a possible way to deal with this to make pattern recognition, multivariate analysis and compound identification better, faster and easier. For a recent review of the subject of machine learning in mass spectrometry imaging applications, see Verbeeck et al. [162].

Finally, it is important to note that no one MSI technique is likely to ever be suitable to image all chemicals in a plant sample. Hence, to achieve a more conclusive and broader picture, MSI techniques should be used in a multimodal fashion in combination with other chemical imaging approaches or with other metabolomic approaches such as LC-MS to provide complementary data and validation [58,163].

## Figures and Tables

**Table 1:** MSI methodologies available for analysis of plant samples.

MSI Methodology	Ionisation Method	Typical Spatial Resolution	Pressure Conditions	Depth Profiling	Drawbacks
**MALDI**	Laser	5–50 µm	Vacuum *	-	Interference of matrix signals. Matrix crystal size restrictions on spatial resolution.
**SIMS**	Primary ions	50 nm–10 μm	Vacuum	yes	Possible fragmentation.
**DESI**	Electrospray	50–200 μm	Ambient	-	Unfavourable for non-polar molecules.
**LAESI**	Laser + electrospray	150–200 μm	Ambient	yes	Low spatial resolution.

*Although typically performed in high vacuum, low vacuum and atmospheric pressure MALDI sources are also available.

**Table 2:** MSI studies to image crop-protection products in plant vegetative structures.

MSI Technique	Plant Species	Agrochemical	Type of Uptake	Reference
**MALDI**	Soy	Mesotrione (Herbicide) Azoxystrobin (Fungicide)	Leaf and root	[59]
**MALDI**	Sunflower	Nicosulfron (Herbicide)	Leaf and root	[57]
**MALDI**	Wheat	Epoxiconazole Azoxystrobin Pyraclostrobin (All fungicides)	Leaf	[56]
**MALDI**	Tomato	Metalaxyl (Fungicide)	Root	[58]
**DESI**	*Cotoneaster horizontalis* *Kalanhoe blossfeldiana*	Rapeseed oil and pyrethrins Imidacloprid and Methiocarb Dimethoate (All insecticides)	Leaf Leaf Root	[133]
**LA-APCI**	Tomato	Isotianil (Fungicide)	Leaf	[137]
**Nano-PALDI**	Flowering cabbage	Chlorantraniliprole (Insecticide)	Leaf	[52]

## Data Availability

Not applicable.
